# Plant–plant communication and community of herbivores on tall goldenrod

**DOI:** 10.1002/ece3.7575

**Published:** 2021-05-02

**Authors:** Kaori Shiojiri, Satomi Ishizaki, Yoshino Ando

**Affiliations:** ^1^ Department of Agriculture Ryukoku University Otsu Japan; ^2^ Department of Natural Environmental Science Niigata University Niigata Japan; ^3^ Field Science Center for Northern Biosphere Hokkaido University Sapporo Japan

**Keywords:** arthropods community, genotypes, goldenrod, plant communication, volatiles

## Abstract

1. The volatiles from damaged plants induce defense in neighboring plants. The phenomenon is called plant–plant communication, plant talk, or plant eavesdropping. Plant–plant communication has been reported to be stronger between kin plants than genetically far plants in sagebrush.
2. Why do plants distinguish volatiles from kin or genetically far plants? We hypothesize that plants respond only to important conditions; the induced defense is not free of cost for the plant. To clarify the hypothesis, we conducted experiments and investigations using goldenrod of four different genotypes.
3. The arthropod community on tall goldenrods were different among four genotypes. The response to volatiles was stronger from genetically close plants to the emitter than from genetically distant plants from the emitter. The volatiles from each genotype of goldenrods were different; and they were categorized accordingly. Moreover, the arthropod community on each genotype of goldenrods were different.
4. *Synthesis*: Our results support the hypothesis: Goldenrods respond to volatiles from genetically close plants because they would have similar arthropod species. These results are important clues elucidating adaptive significance of plant–plant communication.

​

## Graphical abstract

Why do plants distinguish volatiles from kin or genetically far plants? We hypothesize that plants respond only to important conditions; the induced defense is not free of cost for the plant. Our results support the hypothesis: goldenrods respond to volatiles from genetically close plants because they would have similar arthropod species.

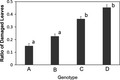

## 1 INTRODUCTION

Because plants are sessile, they must adjust to new growing conditions by detecting and responding to changes in surrounding environment. Plants are known to respond to abiotic environment, such as water stress (Chaves, 2002; Hsiao, 1973; Jaleel et al., 2009), light environment (Demmig‐Adams & Adams, 1992), or temperature change (Buntgen et al., 2015; Levitt, 1980). They also sense and respond to changes in neighboring biotic environment, such as the presence of herbivores and competitors. Upon detection of herbivory, plants may induce resistance to herbivores to minimize further damage. This induced resistance, in contrast to constitutive production of defense, is thought to be a cost‐saving mechanism under infrequent and unpredictable herbivory (Karban & Baldwin, 1997).

Plants may sense the presence of herbivores in the community prior to the actual damage using volatile communication, and thereby prime themselves for future attack. For example, *Arabidopsis thaliana* induces defense gene expression and increases resistance to insect herbivores when they are exposed to plant volatile organic compounds (VOCs) from the neighboring plants (Bate & Rothstein, 1998; Kishimoto et al., 2005). Such plant–plant communication has been reported in more than 30 plant species so far (Heil & Karban, 2010).

Recent studies suggest that communication among plants can be specific: Sagebrush (*Artemisia tridentate*) distinguishes volatiles from self‐ and non‐self‐genotype. The plants which received volatiles from self‐genotype got less damage than the plants which received volatiles from non‐self‐genotype (Karban & Shiojiri, 2009). Moreover, when they received volatiles from genetically closer individuals, they became more resistant than when they received volatiles from genetically distant individuals (Karban et al., 2013). It has been reported that the similarity in the blend of volatiles is related to genetic similarity (Ishizaki et al., 2012). Goldenrod (*Solidago altissima* L) also responds to self‐genotype stronger than non‐self‐genotype by volatiles under the low herbivore population (Kalske et al., 2019). Thus, plants may be able to perceive and respond to volatiles that are similar of their own.

Why should such specificity of plant signaling and communication evolve? Induced plant response is thought as one of the plant's strategies to save defense cost (Agrawal et al., 1999). Plant communication, the response to volatiles of damaged neighboring plants to become resistant to herbivore, is one of the induced plant responses. The merit of plant communication is to be able to induce defense before plants get damage. Kalske et al. (2019) demonstrated that goldenrods that experienced high pressure by herbivory induced resistance in all neighboring conspecifics by volatiles, whereas those experiencing herbivore exclusion induced resistance only in neighbors of the same genotype. Plants would adapt to respond to necessary information. Previous studies indicate that genetically related individuals are similar in leaf chemistry and thus share similar herbivore communities (Kagiya et al., 2018). VOC signals from close relatives could provide accurate information about future herbivory on the receiver plant, whereas VOCs from distantly related individuals may provide misleading information. If there is correlation between similarity of plant genetic and similarity of herbivore community, the receiver plant, tuning into VOC signals from close relatives, is predicted to be more beneficial than that from unrelated individuals.

To test this hypothesis, we conducted these surveys and analysis using tall goldenrods (*Solidago altissima*) as the first step. (1) Do tall goldenrods become more resistant when the plant receives from volatiles from closer genetic plant than from genetically far plant? (2) Are the volatiles different among genotypes, if tall goldenrods can distinguish among genotypes? (3) Are the arthropod community different among genotypes? Are there any relationships between plant genetic similarity and the herbivore community? Finally, we will discuss the beneficial of plant communication in consideration of these results.

## 2 MATERIAL AND METHODS

### 2.1 Study system

Tall goldenrod, *Solidago altissima* L. (Asteraceae), which was introduced to Japan from North America around 1900, is a dominant and well‐studied perennial herb found throughout Japan. Tall goldenrod is host to diverse arthropod communities (Ando et al., 2011). It is rhizomatous and its clones exhibit considerable interclonal genetic variation in many plant traits (Abrahamson & Weis, 1997; Crutsinger et al., 2006, 2008; Maddox & Root, 1987).

In early May 2008, rhizomes of tall goldenrod were collected four genotypes from four sites (one genotype per site) 4.5–17.5 km apart in Shiga Prefecture (Table 1). Rhizomes directly attached to one another were considered as the same genotype. We propagated clones of each genotype from rhizome cuttings into 7 cm in a greenhouse in order to prevent herbivores from feeding. Watering as needed, four genotypes were kept in a large cage until our experiments in 2008, 2011, and 2012.

**TABLE 1:** Original site of each genotype

Genotype	Latitude	Longitude
A	35.04	136.04
B	35.05	136.02
C	35.19	136.08
D	35.06	136.04

### 2.2 Field experiments

#### 2.2.1 Herbivore community census

In early May 2008, 10 rhizome cuttings from each of four genotypes (total of 40 ramets) were individually planted in pots (ca.18 cm, hight 20 cm) and were grown in the large cage until late May. All potted plants were then randomly transplanted into an experimental plot in a 6 m × 16 m grid in the common garden.

The field survey was conducted in our study site at the Center for Ecological Research, Kyoto University, in Otsu, Shiga Prefecture, Japan. To examine how herbivorous insects respond to different genotypes, we conducted herbivore community censuses three times in June 2008. Abundance of each herbivorous insect species was recorded. The density of each arthropod species per plant was calculated by averaging the three census data.

#### 2.2.2 Plant communication experiment

We conducted the field experiments for 2 years at our study site. In the first year (2011), we compared the effectiveness of communication between plants of the self‐ and non‐self‐genotypes. Each set had one potted emitter plant (genotype A) in the center and four potted receiver plants (genotypes A, B, C, and D) around the emitter plant in 2011. Emitter plant was only genotype A, and receiver plants were genotypes A, B, C, and D for all 30 sets. We removed half of each leaf from 25% of the emitter distal leaves with scissors on 29th June. We made thirty sets, communication between an emitter plant and four receivers. We counted the number of leaves with any visible damage caused by herbivores on receiver plants on 10th August, because main damage by herbivorous insects was by August. We also counted the number of all leaves.

In following year (2012), we set up a new experiment to measure the number of natural damages on tall goldenrod unexposed to volatile for each genotype as control, in order to confirm equal damage rate of each genotype. Twelve plants from each genotype set up individually on 20th June in the same field and counted the number of damaged leaves of receiver plants on 7th November as control. Unfortunately, we did not have genotype D because of artificial mistakes.

### 2.3 Genetic dissimilarity of tall goldenrods

To assess genetic dissimilarity among four genotypes, we extracted DNA from green leaf tissue of each genotype using the CTAB method (Milligan, 1992). Following protocols of supporting online material in Crutsinger et al. (2006), we assessed genetic variation among four genotypes by using the AFLP (amplified fragment length polymorphisms) technique (Vos et al., 1995). AFLP markers were generated by using four selective primer pairs: EcoRI‐AGT and MseI‐CTA, EcoRI‐AGT and MseI‐CTT, EcoRI‐AGT and MseI‐CTC, EcoRI‐ACA and MseI‐CTA, and EcoRI‐ACA and MseI‐CTT, and EcoRI‐ACA and MseI‐CTC. Amplicons were separated by ABI PRISM 3130 genetic analyzer (Applied Biosystems). GeneScan was used to visualize AFLP bands. We scored the presence and absence of 113 AFLP amplicons for four genotypes. Genetic distance among genotypes was calculated by Nei's genetic distance (Nei, 1972, 1978), using POPGENE 1.31 (Yeh et al., 1999).

### 2.4 Volatiles collection and analysis

VOCs from artificially damaged tall goldenrods were collected. We planted five tall goldenrods of each genotype in a laboratory room (16L8D, 24 ± 1°C) for around 1 month. We damaged three leaves of each plant with scissors. VOCs from one damaged plant were collected in a glass container (2 L) using Tenax 60/80 (Gerstel GmbH & Co. KG, Mülheim an der Ruhr, Germany) in a laboratory room (24 ± 1°C, light intensity of 6,500 lux) for 30 min. We collected volatiles from each plant as there were five plants for each genotype. Clean air flowed through the glass bottles, and VOCs from the headspace of the bottle were collected at a flow rate of 100 ml/min. n‐Tridecane (0.1 μg), infiltrated onto a piece of filter paper (1 cm^2^), was added as internal standard to the glass container at the onset of VOC collection.

The collected volatile compounds were analyzed by gas chromatograph–mass spectrometer (GC‐MS) (GC: Agilent Technologies; 6890 with HP‐5MS capillary column: 30 m long, 0.25 mm I.D., and 0.25 µm film thickness; MS: Agilent Technologies, 5973 mass selective detector, 70 eV) equipped with a thermal desorption system, a cooled injection system, and a cold trap system (Gerstel GmbH & Co. KG). The headspace volatiles were identified and quantified by comparing their mass spectra and retention times with those of authentic compounds (see above). Quantification of each compound was carried out on the basis of their GC peak areas and expressed as percentages in the total ion chromatogram.

### 2.5 Statistical analyses

#### 2.5.1 Variation in herbivore community among genotypes

To examine whether herbivore community differ between the treatments, we used nonmetric multidimensional scaling analysis (NMDS) with the Bray–Curtis dissimilarity coefficients. Points that are close together represent samples that are very similar in community composition, based on the number of species and relative abundance of each species. Then, difference in community compositions of herbivores among plant genotypes was determined using permutational multivariate analysis of variance (PERMANOVA, Anderson, 2001). Significance was assessed with 999 permutations and the Bray–Curtis dissimilarity. We conducted NMDS and PERMANOVA analysis in MASS and vegan packages of the software R Studio ver. 1.1.383 (R Development Core Team, 2017).

#### 2.5.2 Comparison of herbivore damaged leaves after communication

To compare the ratio of damaged leaves on each genotype, we conducted one‐way ANOVA and after that we used Tukey–Kramer test (JMP 14.2.0) after arcsine transformed. Because the number of the total leaves was different among plants, we used the ratio of damaged leaves instead of number of damaged leaves.

#### 2.5.3 Relationship between plant genetic dissimilarity and the herbivore community

We examined the hypothesis that genotypes that are more genetically similar support more similar herbivore communities, by estimating the pairwise distances between all 40 plants. The correlation between the Bray–Curtis dissimilarity matrix of herbivore communities and Nei's genetic distance matrix, between all the plants (1,560 dataset) was then assessed with a Mantel test (Spearman's rank correlation, 999 permutations). Mantel tests are multivariate measures that evaluate the null hypothesis of no relationship between two similarity matrices. We also conducted a pairwise Mantel test (Spearman's rank correlation, 999 permutations) between genotypes using data averaged on each genotype (6 pairwise data). Mantel tests were performed with XLSTAT version 2010.5.02 (Addinsoft SARL, Paris, France).

#### 2.5.4 Plant volatile compounds relevant to clonal identification

To identify the volatiles compounds that are related to clonal identification, we conducted discriminant analysis (DA) to detect the differences in composition ratio of each compound among genotypes.

However, our volatile profile data included variables whose number (40 compounds) are more than the number of observations (20 individuals) and some of volatile compounds were highly correlated. These situations did not fulfill the condition of DA. Therefore, before conducting DA we transformed the data using principal component analysis (PCA). This procedure allowed us to perform DA with the variables that are uncorrelated and that their number is less than analyzed individuals (Jombart et al., 2010). PCA was performed using prcomp function in stats package of R ver. 3.5.2 (R development core Team 2018). We chose seven principal components that explained 90.2% of variance to submit DA (Table S1). DA was performed using lda function in MASS package. Leave‐one‐out cross‐validation by using CV option of lda function was used to calculate error rate. Error rate was calculated by the number of misclassified samples divided by the total number of samples. The contributions of each compound to linear discriminants were calculated as the sum of products of coefficients of linear discriminant and principal components loadings of each volatiles.

## 3 RESULTS

### 3.1 Herbivore community

**FIGURE 1:**
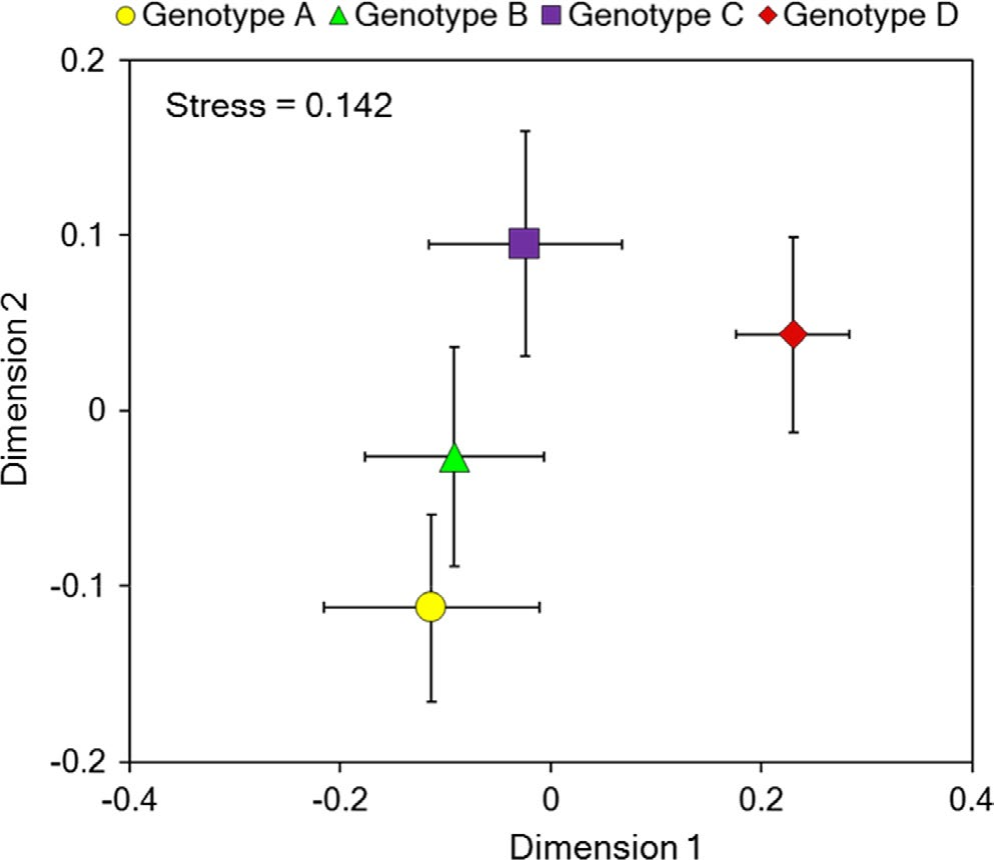
Nonmetric multidimensional scaling (NMDS) ordination of herbivore insect communities on four genotypes of tall goldenrods. The herbivore communities were significantly different among genotypes. Each symbol indicates the mean (±*SE*) of the herbivore community on each genotype

We recorded five herbivorous insect species in four orders on tall goldenrods in June (Table S1). The herbivore community consisted of one Coleoptera (Erateridae sp.), one Diptera (Agromyzidae sp.), two Hemiptera (*Uroleucon nigrotuberculatum*, *Corythucha marmorata*), and one Lepidoptera (*Ascotis selenaria*). The main leaf chewers were a geometrid moth caterpillar, *A*. *selenaria* NMDS analysis of the dissimilarity of herbivore community composition revealed that herbivore community was clearly distinct among four genotypes (Figure 1; PERMANOVA: *R* ^2^ = .19 *p* = .01). NMDS showed that herbivore communities between genotype A and genotype B were the most similar pairs of the four genotypes.

### 3.2 Plant resistance after receiving volatiles in the field

**FIGURE 2:**
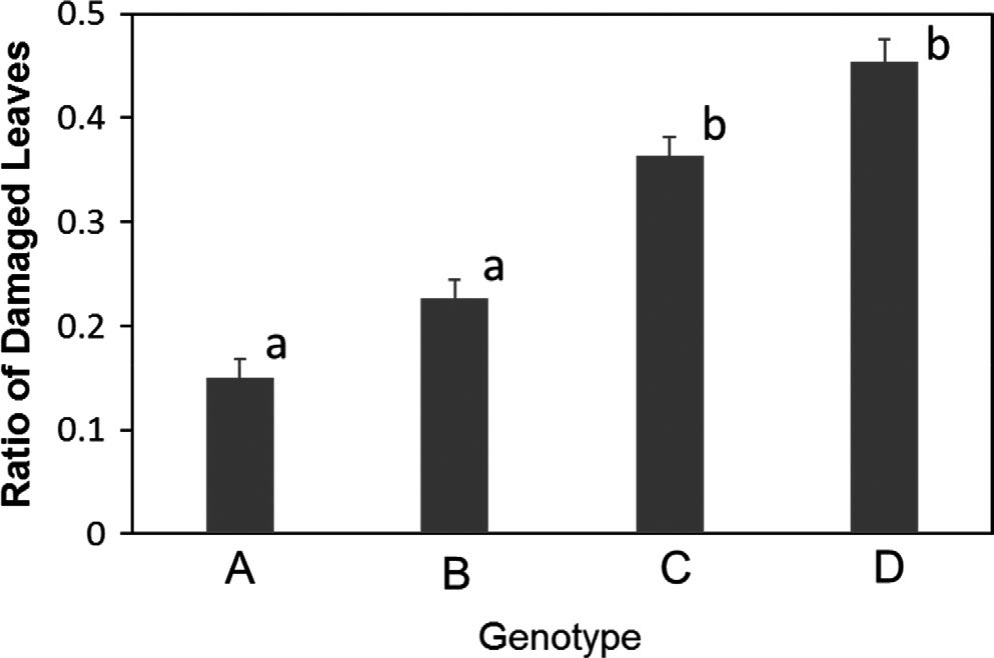
Ratio of damaged leaves of goldenrods in each genotypes. Genotype A was as an emitter

Tall goldenrod plants that received volatiles from the same genotype experienced less damage than other plants. In 2011 when the emitter was genotype A, leaf damage was the lowest on genotype A receiver. The greatest damage was found in genotype D with 45% of leaves damaged by herbivores; more than 2 times as high damage as genotype A (Figure 2). In control (2012), in which the emitter plants were not damaged, the natural damaged leaves were similar among the genotypes in 2012 (*p* = .932, *df* = 2, *F* = 0.145 one‐way ANOVA). The average of damage was 0.10 + 0.02.

### 3.3 Genetic dissimilarity of tall goldenrods

In 59 loci for four genotypes, the number of polymorphic loci was 39. Mean genetic distance between genotypes was 0.40 (range: from 0.29 to 0.49). The most similar genetic distance was between genotype A and genotype B (Table 2).

**TABLE 2:** Genetic dissimilarity of tall goldenrods

	A	B	C	D
A	*			
B	0.2933	*		
C	0.3640	0.3399	*	
D	0.4666	0.4940	0.4140	*

### 3.4 Relationship between plant genetic dissimilarity and the herbivore community

**FIGURE 3:**
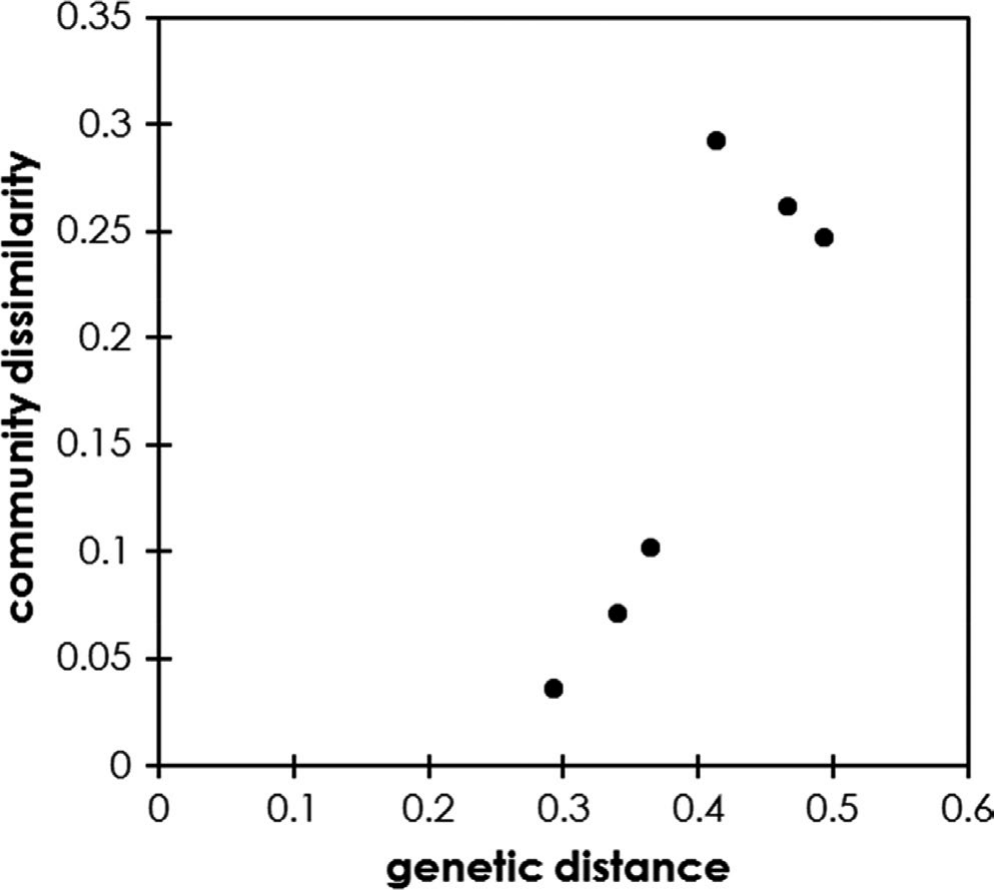
Relationship between genetic distance and community dissimilarity. Plots describe pairwise Mantel correlation comparing distance matrices summarizing herbivore community variation (Bray–Curtis dissimilarity) with those for Nei's genetic distance (Table 2)

The Mantel test between all the plants and pairwise Mantel test between genotypes on community dissimilarity × Nei's genetic distance matrix indicated that genetically related genotype pairs have similar herbivore community (between all the plants: *r* = .11, *p* = .01; between genotypes: *r* = .88, *p* = .001) (Figure 3).

### 3.5 Volatiles from four genotypes

The volatiles from tall goldenrods were comprised of 40 compounds including four unidentified ones (Table 3). Because the amount of volatiles were similar, we compared the ratio of compounds among four genotypes.

**TABLE 3:** Means ± *SD*s of composition ratio (% to total GC peak areas) of each volatile compound detected from each genotype of *Solidago altissima*

Volatile compound	Composition ratio (% to total GC peak areas)			
	Genotype A	Genotype B	Genotype C	Genotype D
Cyclohexane	1.24 ± 1.14	1.59 ± 0.45	1.19 ± 0.7	1.95 ± 1.5
1‐methoxy‐2‐propoxy.ethane	9.16 ± 3.39	3.72 ± 3.72	4.36 ± 4.47	9.47 ± 4.41
2‐Hexenal(E)	0.35 ± 0.79	0.25 ± 0.56	4.08 ± 3.94	2.03 ± 1.98
3‐Hexenol‐1‐ol	5.37 ± 1.87	3.97 ± 2.31	3.62 ± 3.77	3.83 ± 1.39
alpha‐Thujene	0.21 ± 0.47	0.57 ± 1.01	1.13 ± 0.85	1.05 ± 0.88
Alpha‐Pinene	6.19 ± 1	3.63 ± 1.78	8.23 ± 2.68	3.42 ± 0.67
Camphene	1.36 ± 1.2	1.82 ± 3.02	1.2 ± 1.34	0 ± 0
Sabinene	1.8 ± 1.97	3.08 ± 2.05	4.58 ± 4.09	5.16 ± 3.58
2‐Beta‐Pinene	4.32 ± 1.08	1.16 ± 0.91	3.91 ± 2.02	0.75 ± 0.43
Beta‐Myrcene	5.94 ± 0.89	6.04 ± 1.71	6.6 ± 2.39	7.66 ± 2.13
1‐Phellandrene	0.98 ± 0.67	0.82 ± 0.91	0.65 ± 0.91	1.06 ± 0.67
3‐Hexen‐1‐ol,acetate	19.24 ± 5.03	10.67 ± 4.48	17.58 ± 7.91	17.84 ± 11.81
alpha‐Terpinene	1.08 ± 1.24	3.59 ± 2.34	2.75 ± 2.95	3.21 ± 1.81
dl‐Limonene	11.75 ± 4.06	9.56 ± 2.47	13.26 ± 3.78	10.54 ± 2.72
Cyclohexane.1‐methylene‐4	2.87 ± 3.96	0.72 ± 1.61	0.93 ± 2.08	0 ± 0
1.3.6‐Octatriene	0.18 ± 0.41	1.89 ± 0.59	1.28 ± 1.02	1.53 ± 0.92
gamma‐Terpinene	1.6 ± 0.3	3.08 ± 1.83	3.77 ± 2.02	4.34 ± 1.17
alpha‐Terpinolene	0.18 ± 0.4	2.3 ± 1.64	1.7 ± 1.23	1.8 ± 1.12
Nonanal	0.53 ± 0.74	0.41 ± 0.4	0.22 ± 0.49	0.56 ± 0.55
(E)‐4.8‐Dimethyl‐1.3.7‐nonatriene	0.45 ± 0.62	0 ± 0	0 ± 0	0 ± 0
Decanal	1.33 ± 0.88	0.47 ± 0.54	0.32 ± 0.72	1 ± 0.71
Bicyclo2.2.1heptan‐2‐ol	3.05 ± 0.85	1.35 ± 0.92	1.07 ± 1	0.45 ± 0.46
gamma‐Gurjunene	0 ± 0	0.54 ± 0.31	0 ± 0	0 ± 0
Unknown 1	0 ± 0	0.92 ± 0.57	0 ± 0	0.18 ± 0.4
alpha‐Cubebene	1.99 ± 1.82	0 ± 0	0 ± 0	0 ± 0
alpha‐Ylangene	0 ± 0	0.73 ± 0.46	0 ± 0	0.66 ± 0.64
alpha‐Copaene	0 ± 0	1.31 ± 0.8	0.28 ± 0.63	0.66 ± 0.63
Alpha‐Bourbonene	0 ± 0	0.74 ± 0.71	0 ± 0	0.41 ± 0.6
Beta‐Bourbonene	0 ± 0	2.02 ± 0.79	0.7 ± 0.67	1.31 ± 0.84
Cedrene‐V6	0.21 ± 0.48	1 ± 0.3	0.13 ± 0.29	0.62 ± 0.61
Unknown 2	0 ± 0	7.05 ± 1.07	3.39 ± 0.97	0 ± 0
trans‐Caryophyllene	5.2 ± 3.79	0 ± 0	0 ± 0	3.38 ± 3.88
Beta‐Guaiene	0 ± 0	0 ± 0	0 ± 0	0.96 ± 2.14
beta‐Cubebene	0.63 ± 0.9	2.09 ± 1.02	1.07 ± 0.68	1.18 ± 1.09
alpha‐Amorphene	2.87 ± 1.04	2.8 ± 1.25	1.49 ± 1.09	2.83 ± 2.07
Germacrene‐D	4.26 ± 3.27	4.38 ± 3.36	3.42 ± 3.26	1.94 ± 1.35
Isoledene	0 ± 0	6.5 ± 1.29	3.19 ± 1.61	0 ± 0
alpha‐Muurolene	0 ± 0	3.91 ± 1.62	1.86 ± 1.09	3.1 ± 3.45
delta‐Cadinene	4.64 ± 2.05	3.09 ± 1.92	1.25 ± 1.18	3.28 ± 2.45
alpha‐Cadinene	1.01 ± 1.09	2.24 ± 0.62	0.78 ± 0.72	1.87 ± 1.47

**FIGURE 4:**
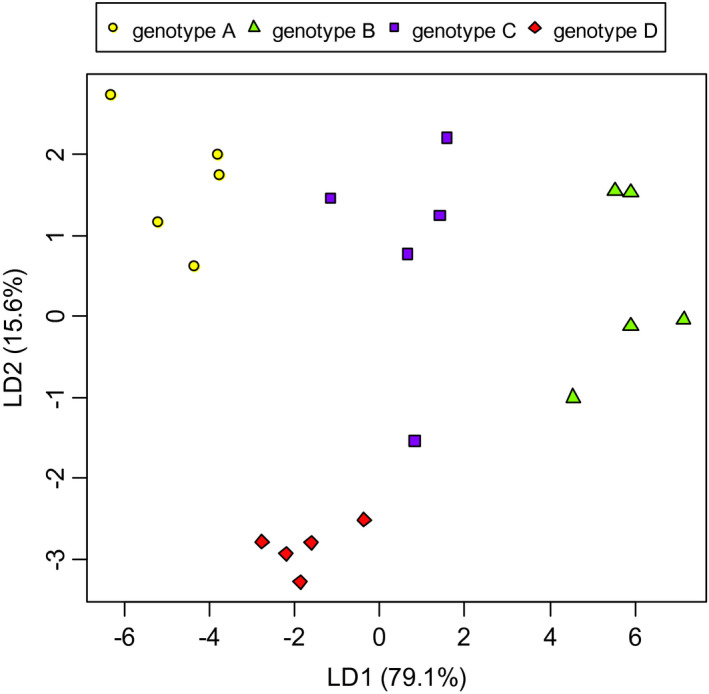
Scatterplot for scores of volatile compounds from four genotypes of *Solidago altissima* based on the first two discriminant functions. Proportion of variance explained by each function are shown in parentheses. Before discriminant analysis, volatile data were transformed to 7 principal components

Volatile profiles from different genotypes were profoundly different, while that of the same genotype were similar. Twenty‐five compounds of 40 volatile compounds were emitted from all genotypes, while the others were not found in one or more genotypes (Table 3). Discriminant analysis revealed that first and second discriminant functions explained 79.1% and 15.6% of variance, respectively (Table 4), and showed clear discriminations of genotypes (Figure 4), with the error rate 0.15. First discriminant function was mainly contributed by PC2 and PC4, and second discriminant function was contributed by PC3 and PC7 (Table 4). First discriminant function which was positively contributed by γ‐Gurjunene, unknown 2, and Isoledon discriminated genotype B that emitted those 3 compounds highly (Tables 3 and 5, Figure 4). Second discriminant function discriminated genotype D that emitted less 2‐β‐Pinene and Bicyclo2.2.1heptan‐2‐ol, and more γ‐Terpinene than other genotypes (Tables 3 and 5, Figure 4).

**TABLE 4:** Results of discriminant analysis for 7 principal components (PCs)

	LD1	LD2
Proportion of trace	0.791	0.156
Coefficients of linear discriminants:		
PC1	23.11	0.465
PC2	**−51.746**	−4.47
PC3	−1.823	**26.356**
PC4	**−35.979**	18.513
PC5	**29.444**	**26.282**
PC6	4.448	−12.774
PC7	15.729	**−28.696**

Note

PCs with strong coefficient (first three strongest) on a giving linear discriminant function (LD) are shown in bold.

**TABLE 5:** Contributions of each volatile compound to linear discriminant functions

	LD1	LD2
Cyclohexane	16.640	−10.557
1‐methoxy‐2‐propoxy.ethane	−23.522	−10.034
2‐Hexenal(E)	−13.291	−7.914
3‐Hexenol‐1‐ol	−8.599	10.321
alpha‐Thujene	−8.987	−18.584
Alpha‐Pinene	−21.457	25.078
Camphene	4.976	26.761
Sabinene	4.497	−19.820
2‐Beta‐Pinene	−35.621	**38.629**
Beta‐Myrcene	−9.986	−19.063
1‐Phellandrene	−18.901	−1.388
3‐Hexen‐1‐ol,acetate	−12.731	−0.167
alpha‐Terpinene	16.329	−14.152
dl‐Limonene	−2.561	7.321
Cyclohexane.1‐methylene‐4	−25.604	24.764
1.3.6‐Octatriene	31.947	−20.522
gamma‐Terpinene	4.730	**−29.605**
alpha‐Terpinolene	21.079	−12.401
Nonanal	−8.270	−11.140
(E)‐4.8‐Dimethyl‐1.3.7‐nonatriene	−27.578	11.671
Decanal	−32.025	1.590
Bicyclo2.2.1heptan‐2‐ol	−26.348	**33.497**
gamma‐Gurjunene	**50.783**	1.021
Unknown 1	38.825	−8.134
alpha‐Cubebene	−40.062	20.606
alpha‐Ylangene	32.304	−18.811
alpha‐Copaene	44.439	−2.863
Alpha‐Bourbonene	28.669	−4.286
Beta‐Bourbonene	45.125	−18.010
Cedrene‐V6	27.838	−2.852
Unknown 2	**66.484**	12.125
trans‐Caryophyllene	−48.608	−4.608
Beta‐Guaiene	−9.091	−22.092
beta‐Cubebene	34.167	−12.427
alpha‐Amorphene	−10.335	−6.831
Germacrene‐D	7.787	6.783
Isoledene	**64.970**	14.491
alpha‐Muurolene	36.445	−19.767
delta‐Cadinene	−18.922	3.047
alpha‐Cadinene	14.166	−11.449

Note

Contributions were calculated as the sum of products of coefficients of linear discriminant and principal components loadings of each volatiles. Volatile compounds with strong contribution (first three strongest) on a giving linear discriminant function (LD) are shown in bold.

## 4 DISCUSSION

In the field experiments, we showed that tall goldenrod which received volatiles from same genotype got the least damage than the other genotypes. Some plants such as sagebrush (Karban & Shiojiri, 2009), *Ambrosia dumosa* (Mahall & Callaway, 1996), and *Cayratia japonica* (Fukano & Yamawo, 2015) can distinguish between self and non‐self by volatiles. Our result partially supports previous tall goldenrod study in recognizing the same genotype (Kalske et al., 2019). Kalske et al. (2019) showed that plants induced resistance in the same genotype under lower herbivore pressure and in all genotypes under higher herbivore pressure. On the other hand, the goldenrod which received volatiles from closer genotype got less damages in our experiments. The results suggest that the goldenrod could recognize the volatiles of genetically closer plants. As for whether the induction of plant resistance is limited to closer relatives, it may depend on the history of the degree of herbivore pressure. Herbivore pressure in our field is likely to be lower than in the original habitats with many natural enemies (e.g., the enemy‐free hypothesis) (Fukano & Yahara, 2012), so all genotypes did not need to respond to volatiles of damaged leaves in the same way. Kin recognition through volatiles also has been reported in Sagebrush (Karban et al., 2013).

To distinguish volatiles from kin from nonkin in goldenrod, the volatiles should be different among genotype. Actually, volatiles of goldenrod were different between genotypes (Figure 4). Our result of discriminant analysis indicated that a few volatile compounds are associated with clone identification. In our study, γ‐Gurjunene, unknown 2, Isoledon, 2‐β‐Pinene, Bicyclo2.2.1heptan‐2‐ol, and γ‐Terpinene were suggested to contribute to genotype identification (Table 4). More study is needed to clarify whether these compounds actually cause clonal distinction. In our analysis of volatile profile, genotype A and genotype B, which are genetically close, showed considerably different volatile profiles. Therefore, we could not find the correlation between similarities of volatiles and genetics.

Why plants distinguish volatiles information? Because of the cost of induced defense, plants want to respond only to serious information (alarm). There are significant positive correlations between community dissimilarity and neutral molecular genetics in foundation tree species (Barbour et al., 2009). Johnson and Agrawal have demonstrated in evening primrose, *Oenothera biennis* L. (Onagraceae), that genetic variation in plant traits such as plant size, architecture, and reproductive phenology affect arthropod community (Johnson & Agrawal, 2005). In tall goldenrods, the herbivorous communities were significantly different among genotype and the community dissimilarity was correlated with genetic distance (Figures 1 and 3). This means that the herbivore species for plants are different among genotypes, but genetically closer genotypes have a more similar insect community structure, suggesting that future herbivory is more likely to be similar. Plants should not respond to information from far genotypes. They must respond to serious dangers, such as when kin plants are damaged.

The volatiles must be useful information to the neighbor plant. They could predict the level of danger from volatiles’ information. There are at least 15 different compounds in volatiles between these four genotypes. This suggests a clone distinguishing based on difference in blend. We use the volatiles of artificially damaged plants. However, the plants are known to release different blend volatiles depending on different types of damage caused by different herbivores (Aljbory & Chen, 2018). A future work will be to discover whether plants can distinguish among damage varieties.

Although we used only four genotypes, these experiments and survey are the first step for understanding why plants distinguish among volatiles, especially from kin and nonkin. If we used more genotypes from different areas in our experiment, we expect to get the same result with better statistics. In conclusion, our results supported the hypothesis: goldenrods respond to volatiles from close‐genotype plants because they would have similar arthropod species. These results are important clues elucidating adaptive significance of plant–plant communication.

## CONFLICT OF INTEREST

None declared.

## AUTHOR CONTRIBUTION

**Kaori Shiojiri:** Conceptualization (lead); Data curation (equal); Formal analysis (equal); Funding acquisition (lead); Investigation (lead); Methodology (lead); Project administration (lead); Resources (equal); Software (equal); Supervision (equal); Validation (lead); Visualization (equal); Writing‐original draft (lead); Writing‐review & editing (lead). **Satomi Ishizaki:** Conceptualization (supporting); Data curation (equal); Formal analysis (equal); Funding acquisition (equal); Investigation (supporting); Methodology (supporting); Project administration (supporting); Software (equal); Supervision (supporting); Validation (equal); Visualization (equal); Writing‐original draft (supporting); Writing‐review & editing (supporting). **Yoshino Ando:** Conceptualization (supporting); Data curation (equal); Formal analysis (equal); Funding acquisition (supporting); Investigation (supporting); Methodology (supporting); Project administration (supporting); Resources (equal); Software (equal); Supervision (supporting); Validation (equal); Visualization (equal); Writing‐original draft (supporting); Writing‐review & editing (supporting).

## Supporting information

Tables S1, S2

## Data Availability

Data from this manuscript were archived in the publicly accessible repository Dryad (https://doi.org/10.5061/dryad.80gb5mkpv).
