# Supplementary material for: Plant–plant communication and community of herbivores on tall goldenrod
Source: Ecol Evol. 2021 May 2;11(12):7439–47. doi: 10.1002/ece3.7575 (PMC8216902; doi:10.1002/ece3.7575)
Supplement: Supplementary file 1 — Tables S1, S2 [file ECE3-11-7439-s001.docx]

Table S1 Five species of herbivorous insects found on each of 4 genotypes of tall goldenrod.

|  |  |  | Plant genotype^1^ | | | |
| --- | --- | --- | --- | --- | --- | --- |
| Species | Feeding guild | Stage | A | B | C | D |
| Coleoptera |  |  |  |  |  |  |
| Elateridae sp. | leaf chewer | adult | ○ | － | － | － |
|  |  |  |  |  |  |  |
| Diptera |  |  |  |  |  |  |
| Agromyzidae sp. | leaf miner | larva | ○ | ○ | ○ | ○ |
|  |  |  |  |  |  |  |
| Hemiptera |  |  |  |  |  |  |
| *Corythucha marmorata* | sap feeder | nymph, adult | ○ | ○ | ○ | ○ |
| *Uroleucon nigrotuberculatum* | sap feeder | nymph, adult | － | ○ | － | － |
|  |  |  |  |  |  |  |
| Lepidoptera |  |  |  |  |  |  |
| *Ascotis selenaria* | leaf chewer | larva | ○ | ○ | ○ | ○ |

^1^ The herbivore species was observed (○) or not observed (－) on the genotype during the field survey.

Table S2
